# Supplementary material for: pH-Dependent Molecular Gate Mesoporous Microparticles for Biological Control of Giardia intestinalis
Source: Pharmaceutics. 2021 Jan 13;13(1):94. doi: 10.3390/pharmaceutics13010094 (PMC7828499; doi:10.3390/pharmaceutics13010094)
Supplement: Supplementary file 1 [file pharmaceutics-13-00094-s001.pdf]

pharmaceutics

Supplemetary Materials: pH-Dependent Molecular Gate Mesoporous Microparticles for Biological Control of *Giardia intestinalis*

Isabel González-Alvarez, Verónica Vivancos, Carmen Coll, Bárbara Sánchez-Dengra, Elena Aznar, Alejandro Ruiz-Picazo, Marival Bermejo, Félix Sancenón, María Auxiliadora Dea-Ayuela, Marta Gonzalez-Alvarez and Ramón Martínez-Máñez

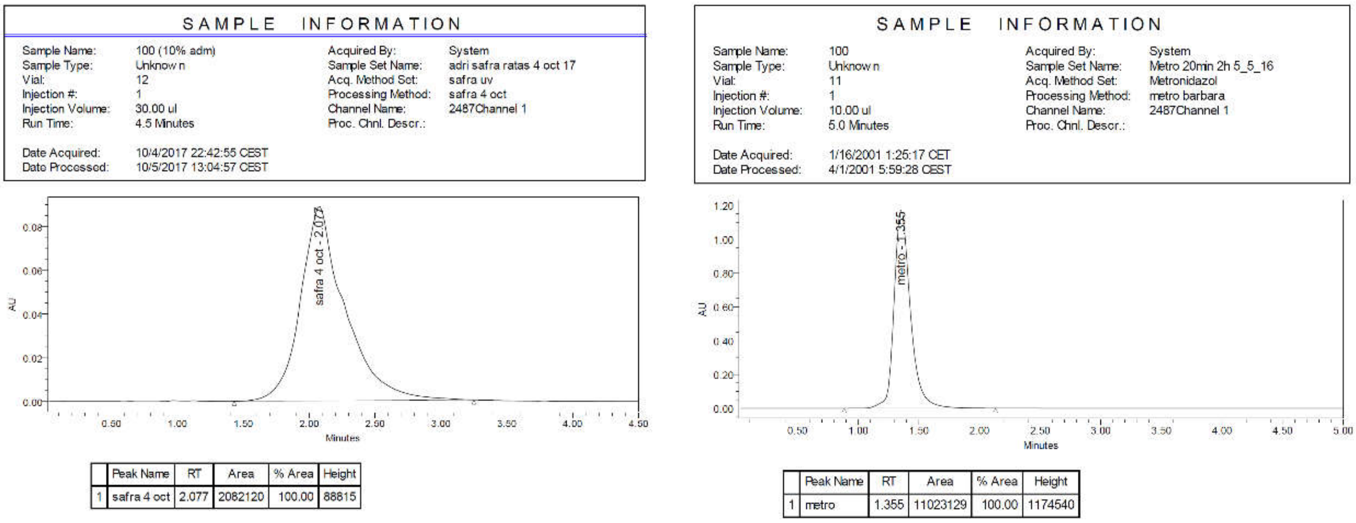

(**a**) (**b**)

Figure S1. HPLC chromatograms of safranine (**a**) and metronidazole (**b**).
